# A potential role for Galectin-3 inhibitors in the treatment of COVID-19

**DOI:** 10.7717/peerj.9392

**Published:** 2020-06-15

**Authors:** John L. Caniglia, Maheedhara R. Guda, Swapna Asuthkar, Andrew J. Tsung, Kiran K. Velpula

**Affiliations:** 1 Department of Cancer Biology and Pharmacology, Neurosurgery, and Pediatrics, University of Illinois College of Medicine at Peoria, Peoria, IL, USA; 2 Departments of Cancer Biology and Pharmacology, Neurosurgery, and Pediatrics, University of Illinois College of Medicine at Peoria, Peoria, IL, USA; 3 Illinois Neurological Institute, Peoria, IL, USA; 4 Departments of Cancer Biology and Pharmacology, Pediatrics, Neurosurgery, and Pediatrics, University of Illinois College of Medicine at Peoria, Peoria, IL, USA

**Keywords:** Galectin-3, Cytokines, COVID-19

## Abstract

The outbreak of severe acute respiratory syndrome coronavirus 2 (SARS-CoV2), the causative agent of coronavirus disease 2019 (COVID-19), has been declared a global pandemic by the World Health Organization. With no standard of care for the treatment of COVID-19, there is an urgent need to identify therapies that may be effective in treatment. Recent evidence has implicated the development of cytokine release syndrome as the major cause of fatality in COVID-19 patients, with elevated levels of interleukin-6 (IL-6) and tumor necrosis factor alpha (TNF-α) observed in patients. Galectin-3 (Gal-3) is an animal lectin that has been implicated in the disease process of a variety of inflammatory conditions. Inhibitors of the small molecule Gal-3 have been shown to reduce the levels of both IL-6 and TNF-α in vitro and have shown anti-inflammatory effects in vivo. Additionally, a key domain in the spike protein of β-coronaviridae, a genus which includes SARS-CoV2, is nearly identical in morphology to human Gal-3. These spike proteins are critical for the virus’ entry into host cells. Here we provide a systematic review of the available literature and an impetus for further research on the use of Gal-3 inhibitors in the treatment of COVID-19. Further, we propose a dual mechanism by which Gal-3 inhibition may be beneficial in the treatment of COVID-19, both suppressing the host inflammatory response and impeding viral attachment to host cells.

## Introduction

With the ongoing pandemic attributed to the pathogenic severe acute respiratory syndrome coronavirus 2 (SARS-CoV2), there is an urgent need to identify effective treatment options (Zhou et al., 2020). Numerous treatments, most notably the antiviral agent remdesivir and the antiparasitic drug chloroquine, have been extensively studied with inconclusive results (Zhai et al., 2020; Ko et al., 2020). The current absence of a proven, effective anti-viral therapy has resulted in anti-inflammatory agents such as interleukin-6 (IL-6) and tumor necrosis factor alpha (TNF-α) inhibitors being proposed to mitigate symptoms (Perricone et al., 2020). The rationale is largely based on the finding that patients requiring intensive care hospitalization showed highly elevated levels of these pro-inflammatory cytokines (Perricone et al., 2020). Cytokine inhibitors are currently in clinical trials and have not yet been proven effective.

The lack of an effective standard of care and rate at which coronavirus disease 2019 (COVID-19) is spreading has accelerated the need to identify novel treatment options. Though prior research has elucidated the structural homology between the spike proteins of β-coronaviruses and human Gal-3, there is no published literature to date referencing this information in the context of SARS-CoV2. Furthermore, there are no articles to date proposing Gal-3 inhibition as a potentially viable treatment to mitigate the entry of SARS-CoV2 and the inflammatory response associated with infection. As such, the authors see a need to spread awareness of the promising indications for targeting Gal-3 in the treatment of COVID-19. This article is intended for all researchers studying galectins and/or SARS-CoV2 but may be particularly beneficial to those focused on drug discovery and development.

## Survey Methodology

### Eligibility criteria

The following is comprised of original studies that provided information about β-coronaviridae, Gal-3, or Gal-3 inhibitors. This article includes results from both in vivo and in vitro studies and this information is specified where applicable to ensure clarity. The following types of studies were excluded: (1) studies with only an abstract or no full-text available; (2) books, conference papers, and theses.

### Search methodology

To retrieve primary literature, electronic searches were performed on PubMed and Google Scholar. A list of search terms can be seen in Table S1. Gal-3 expression was obtained from The Human Protein Atlas with the following query: “Galectin-3 (Gal-3)”. The structures of the SARS-CoV2 S1-NTD and Gal-3 were obtained from the RCSB Protein Data Bank (PDB) using the following queries: “SARS-CoV2 spike protein”; “Gal-3”.

### Risk of bias

To minimize the risk of erroneous conclusions, all authors assessed the cited studies for quality. To address key claims in the article including but not limited to: coronavirus mechanisms of entry, Gal-3 suppression inhibiting IL-6 and TNF-α release, the side effects of Gal-3 inhibition in humans, and the structural similarities of Gal-3 with the S1-NTD of β-coronaviruses, multiple sources were cited throughout to mitigate error. Additionally, the systematic use of open-ended search queries ensured that a comprehensive profile of results was yielded on the subject matter.

### Galectins and the SARS-CoV2 spike protein

Galectins, a structurally similar family of animal lectins with chemokinetic properties, have been implicated extensively in the immune response (Elola et al., 2018). Gal-3, arguably the most well studied of the galectins, has been shown to activate the pro-inflammatory transcription factor NF-kB and induce the release of both IL-6 and TNF-α (Filer et al., 2009; Uchino et al., 2018). Additionally, data obtained from The Human Protein Atlas shows baseline Gal-3 protein expression in healthy tissues is highest in the lungs, followed by the gastrointestinal tract (stomach, duodenum, small intestine, colon and rectum) and brain (cortex and hippocampus). This is particularly noteworthy as an increasing number of patients infected with SARS-CoV2 have reported gastrointestinal symptoms such as diarrhea, nausea, vomiting and abdominal pain (Patel et al., 2020). Additionally, a Mao et al. (2020) study assessed 214 cases of COVID-19 in Wuhan, China and concluded that 36.4% of patients in this cohort exhibited neurological symptoms such as cerebrovascular events, impaired consciousness and muscle injury. Additionally, a systematic review Whittaker, Anson & Harky (2020) reports incidences of headache, seizures, asomnia and an isolated case of Guillan-Barre syndrome that have occurred following SARS-CoV2 infection.

**Figure 1:**
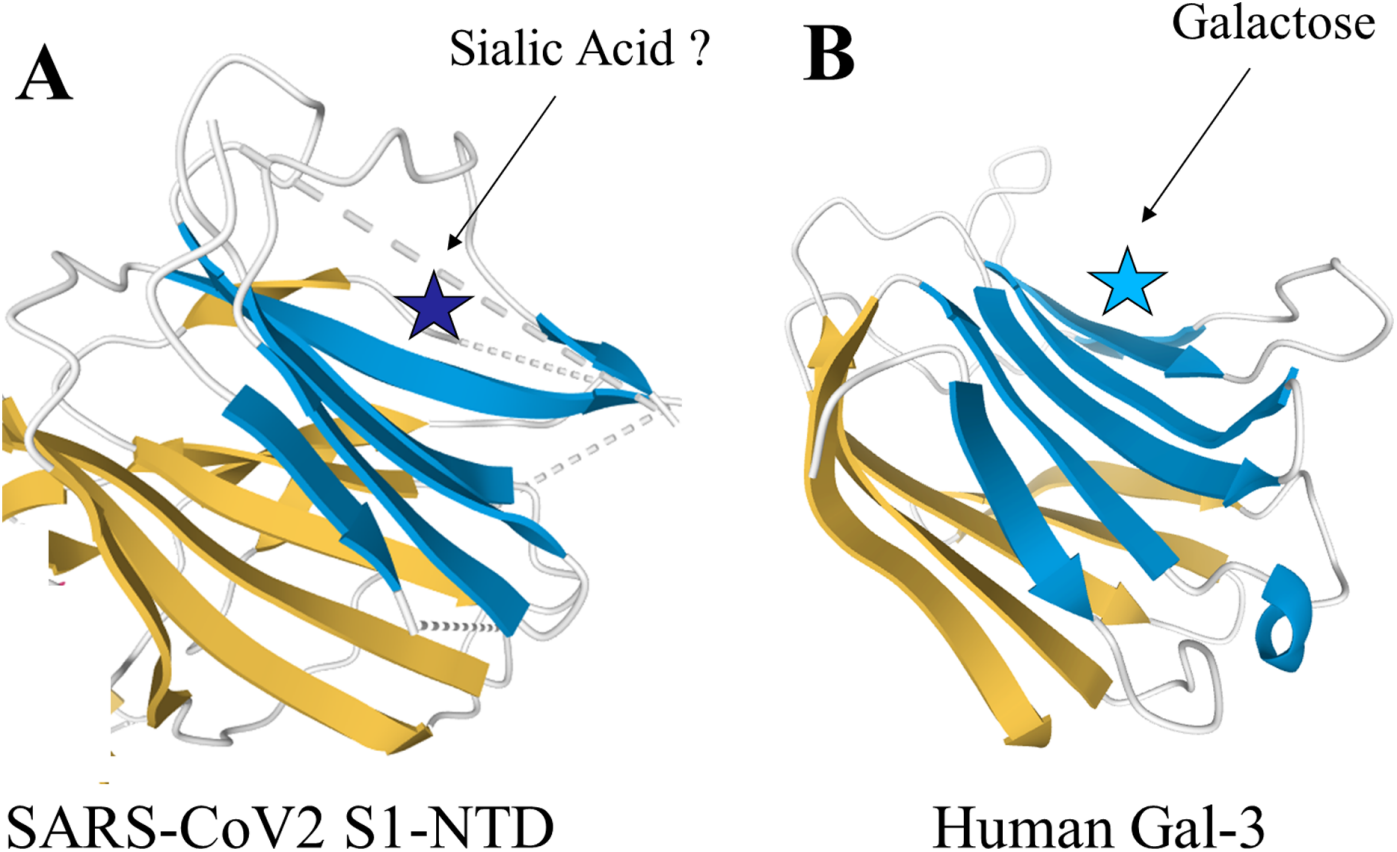
Structural similarities of SARS-CoV2 S1-NTD and human Gal-3. The structural topologies of the (A) SARS-CoV2 S1-NTD (PDB ID: 6VXX) and (B) human Gal-3 (PDB ID: 1A3K) are shown as schematic illustrations, where β-strands are depicted as arrows and α-helices as cylinders.

The spike proteins found in the β-genus of coronaviridae share unique structural similarities with human Gal-3 (Li, 2016). Structural analysis of the N-terminal domain (NTD) of the spike protein subunit S1 in murine hepatitis virus (MHV) showed a nearly identical topology to human Gal-3, with the only difference being two additional β-strands in one of the β-sheet layers of MHV S1-NTD (Li, 2015; Peng et al., 2011) Additionally, a study of bovine coronavirus (BCoV) found significant overlap between the virus’s S1-NTD receptor binding domain and the galactose-binding domain of human galectins, strongly supporting a functional similarity (Peng et al., 2012). Pertinent to these findings is the high degree of structural conservation in the S1-NTD observed amongst the β-genus of coronaviridae, which now includes SARS-CoV2 (Li, 2015). Given this structural similarity, it may be possible that inhibitors against human galectins also have the capability to bind the S1-NTD of β-coronaviridae. Further research will be required to fully examine this hypothesis. Specifically, the intramolecular forces present within the SARS-CoV2 S1-NTD, such as charge–charge interactions and hydrophobicity, and how they compare to human Gal-3 warrants further investigation. The structures of the SARS-CoV2 S1-NTD and human Gal-3 are shown in Fig. 1.

### Coronavirus attachment: significance of the galectin-like S1-NTD

The recognition and binding of membrane-bound cell receptors is the first step in viral infection and a necessary event prior to cell invasion (Blaas, 2016). In β-coronaviridae such as SARS-CoV2, this function is mediated entirely by the S1 subunit of the spike protein (Li, 2016). The S1 subunit can be further divided into two distinct domains: the NTD and the C-terminal domain (CTD) (Wang et al., 2020). Though both domains play a role in the adhesion process, the receptor binding mechanisms amongst these viruses can be thought of as predominantly CTD or NTD mediated. A general rule is that the CTD mainly binds peptides while the NTD mainly binds extracellular sugars, though there are exceptions such as that seen in the entry mechanism of MHV via carcinoembryonic antigen cell adhesion molecule 1 (CEACAM1) (Li, 2015). A pivotal study by Wang et al. (2020) has shown structural evidence that SARS-CoV2 binds to host angiotensin-converting-enzyme-2 (ACE2) receptors in a CTD mediated fashion (Li, 2016). The main binding receptors utilized by each of the β-coronaviridae can be seen in Table 1.

**Table 1:** Receptors used for entry amongst β-coronaviridae.

Virus	Receptor	NTD/CTD mediated
MHV	CEACAM1	NTD mediated
BCoV	Sialic acids	NTD mediated
MERS-CoV	DPP4	CTD mediated
SARS-CoV	ACE2	CTD mediated
SARS-CoV2	ACE2	CTD mediated

As can be seen in Table 1, the strongly related virus BCoV attaches to sialic acids on host cells, specifically 9-*O*-acetyl-sialic acid (9-*O*-Ac-Sia), in an NTD mediated mechanism (Peng et al., 2012). Specifically, members of this species known to infect humans such as human coronavirus OC43 and HKU1 are among the viruses that bind 9-*O*-Ac-Sia (Tortorici et al., 2019). In humans, sialic acids including 9-*O*-Ac-Sia are most present within the body at mucosal surfaces such as the nasopharynx, lungs, and gastrointestinal tract (Barnard et al., 2019).

More pertinent than these findings, however, is the evidence that coronaviruses which bind receptors in a CTD-mediated fashion still are reliant upon their galectin-like NTD for functioning. In a study by Li et al. (2017) it was shown that MERS-CoV, in addition to binding dipeptidyl peptidase 4 (DPP4) through its CTD domain, selectively binds to sialic acids at the NTD domain. Additionally, the depletion of sialic acids through treatment with neuraminidase inhibitors inhibited MERS-CoV entry into Calu-3 human airway cells, demonstrating that sialoconjugate binding by the galectin-like NTD is an essential component of MERS-CoV infection (Li et al., 2017).

**Figure 2:**
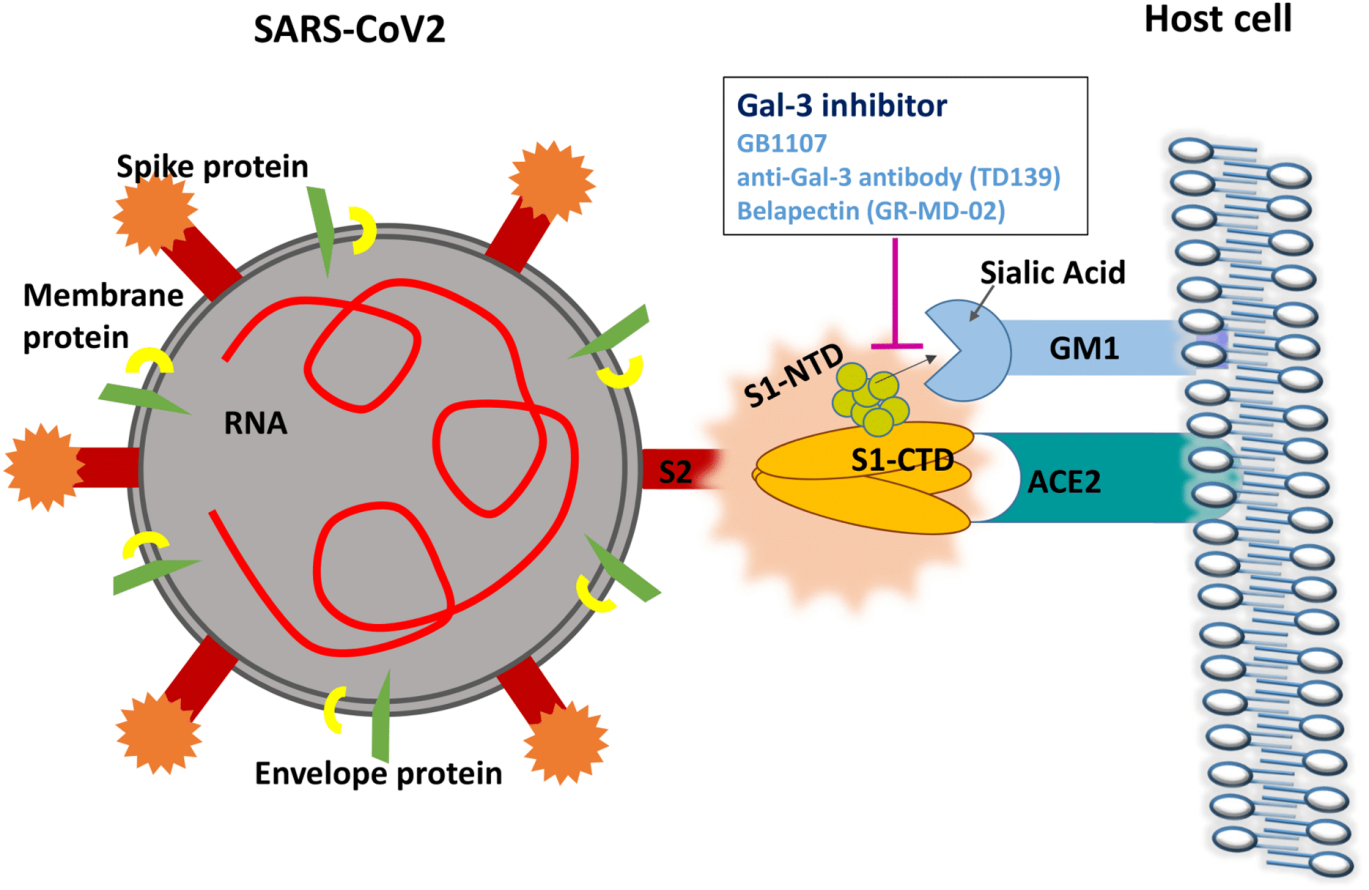
Gal-3 inhibition may disrupt the attachment of SARS-CoV2 S1-NTD to GM1 gangliosides on the cell surface.

To date, there are no studies investigating whether sialic acid binding is an essential component of SARS-CoV2 infection. However, a recent study has elucidated the crystalline structure of the SARS-CoV2 S1-NTD (Ou et al., 2020). Molecular dynamic simulations of the tip of the S1-NTD (amino acids 100–175) reveal a strong interaction with GM1 ganglioside, a molecule commonly found on cell surfaces (Fantini et al., 2020). This data strongly supports a dual attachment model for SARS-CoV2 similar to the mechanism observed in MERS-CoV, where the CTD domain is involved in ACE-2 receptor recognition and the NTD region binds gangliosides on the cell surface to stabilize viral adhesion (Fantini et al., 2020). Human galectins have also been shown to bind GM1 ganglioside with high affinity (Wu et al., 2016). The proposed mechanism by which Gal-3 inhibitors may disrupt SARS-CoV2 attachment is shown in Fig. 2.

### Gal-3 inhibitors in inflammation

**Figure 3:**
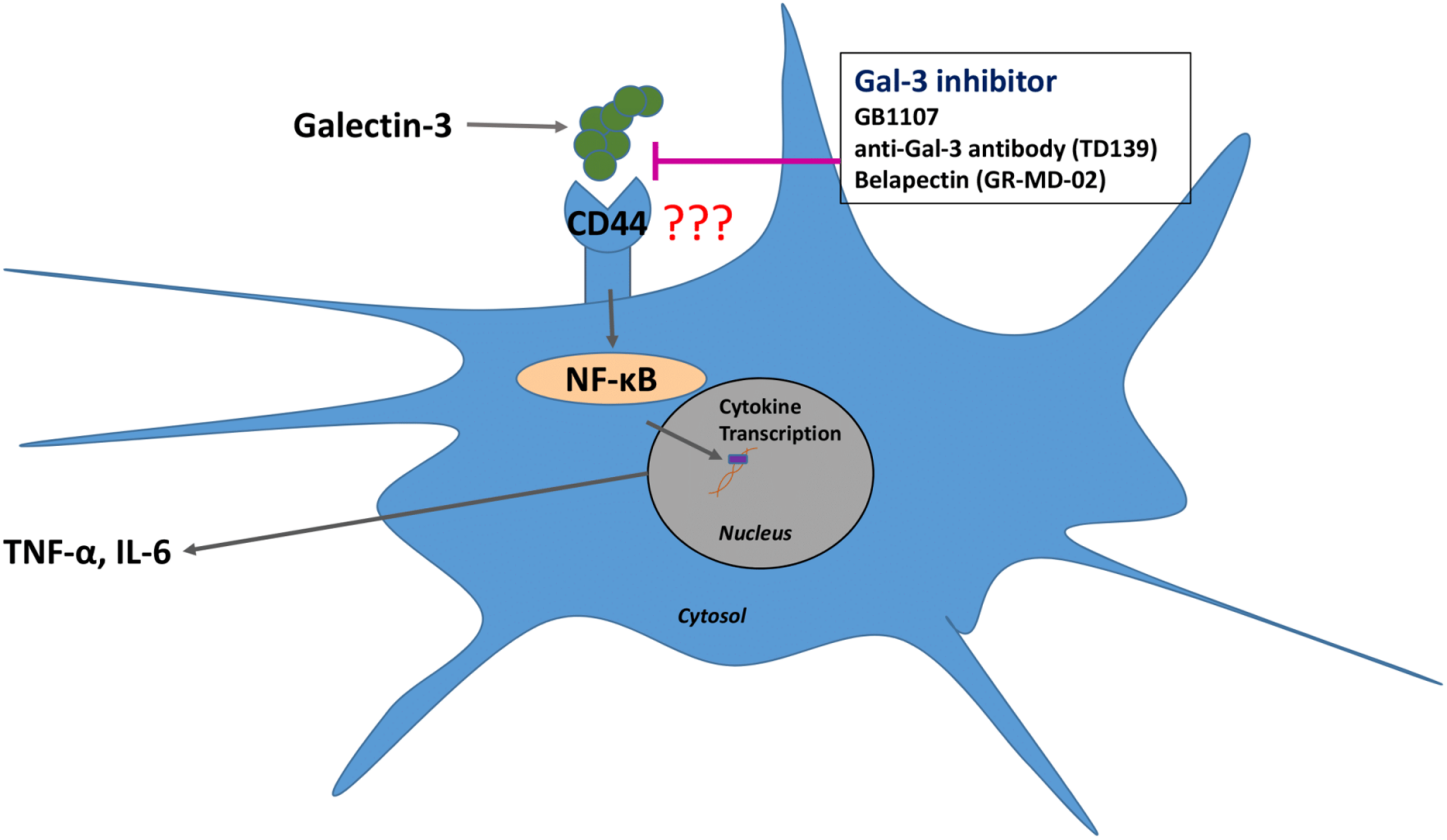
Gal-3 inhibition suppresses the release of IL-6 and TNF-α from dendritic cells.

In addition to Gal-3 inhibition potentially being able to bind and disrupt the NTD of β-coronaviridae, inhibiting Gal-3 has shown numerous anti-inflammatory effects that may be beneficial in the treatment of COVID-19 (Stegmayr et al., 2019). Retrospective studies of the MERS-CoV and SARS-CoV outbreaks have provided evidence that cytokine release syndrome (CRS)-induced pneumonia was the major cause of fatality in affected patients (Channappanavar & Perlman, 2017). In SARS-CoV, the virus efficiently invades monocytes and dendritic cells, inducing the release of pro-inflammatory cytokines such as interleukin 1 (IL-1), IL-6 and TNF-α (Law et al., 2005). Recent evidence has implicated the CRS as a major cause of fatality in COVID-19 patients as well, and this process is likely dendritic cell mediated (Moore & June, 2020). A study by Chen et al. (2015) has shown that Gal-3 inhibition simultaneously reduces the production of inflammatory cytokines such as IL-1 and IL-6 while also increasing the levels of the anti-inflammatory interleukin 10 (IL-10) in human dendritic cells. A reduction in IL-1, IL-6 and TNF-α levels upon treatment with the Gal-3 inhibitor GB1107 was also seen in an inflammatory model of spinal cord injury (Ren et al., 2019). Treatment with Gal-3 inhibitors shows promise in reducing the incidence of CRS in SARS-CoV2 patients through directly suppressing the release of pro-inflammatory cytokines by dendritic cells. Additionally, as increased Gal-3 levels have been shown in virally infected cells, these inhibitors may preferentially bind in highly affected regions of the body (Wang et al., 2019). The proposed mechanism of Gal-3 inhibitors in reducing inflammatory cytokine release can be seen in Fig. 3.

There are studies that have more specifically examined the effects of Gal-3 inhibition as it relates to viral pathogenesis. A study from Chen et al. (2018) has shown that Gal-3 is upregulated in the lungs of mice infected with H5N1 influenza A virus. Compared to controls, Gal-3 K/O mice had a higher survival rate, with 36% of Gal-3 K/O mice surviving infection compared to 0% with Gal-3 expression (Chen et al., 2018). The Gal-3 K/O mice also exhibited significantly lower levels of IL-1β than controls (Chen et al., 2018). Additionally, in a murine study of γ-herpesvirus MHV68, Gal-3 K/O mice mounted a stronger CD8+ T-cell response and showed better viral control compared to controls (Kaur et al., 2018) However, as the role of Gal-3 in regulating the innate immune response is widespread, further studies will be needed to fully determine the systemic effects of Gal-3 inhibition.

There are several Gal-3 inhibitors that have been developed to date, with some currently in clinical trials (Blanchard et al., 2014). The use of the Gal-3 inhibitor TD139 has completed phase I/IIa trials in the treatment of idiopathic pulmonary fibrosis, which showed the drug to be safe and well tolerated (Saito et al., 2019). A phase IIb trial of the drug is ongoing (Saito et al., 2019). Phase II trials of another Gal-3 inhibitor, belapectin (also known as GR-MD-02), showed a significant reduction in portal hypertension and the development of new esophageal varices (EV) in patients with nonalcoholic steatohepatitis (NASH) complicated by EV (Chalasani et al., 2020). Phase III trials of this drug in the treatment of NASH are currently ongoing. It is worth noting that biweekly infusions of belapectin (2 mg/kg) over the course of a year did not result in any serious adverse effects (Chalasani et al., 2020). However, given the role of Gal-3 in immunomodulation, more studies will be needed to fully evaluate the safety of belapectin, as to date it has been administered to just over 3,000 patients. On a similar note, a novel galectin-1 inhibitor is currently being developed for the treatment of COVID-19. The drug may be able to modulate the cytokine storm associated with COVID-19 as well as impede viral attachment. However, further studies will be necessary to fully evaluate the proposed drug’s efficacy in COVID-19 treatment.

## Conclusions

In summary, Gal-3 is a lectin secreted by many types of cells that exhibits potent pro-inflammatory effects. This includes inducing the production of IL-6 and TNF-α, cytokines which have been shown to play a critical role in the development of CRS (Filer et al., 2009; Uchino et al., 2018; Stegmayr et al., 2019). Importantly, the spike proteins utilized by β-coronaviridae show strikingly similar morphology to Gal-3 and exhibit similar sugar-binding capabilities (Li, 2016). Taken together, the strong correlation of organs showing high Gal-3 expression and symptoms of SARS-CoV2, anti-inflammatory effects of Gal-3 inhibition, and theorized ability of galectin inhibitors to impair NTD-mediated viral attachment make Gal-3 an attractive potential target in the treatment of COVID-19 (Li et al., 2017; Moore & June, 2020). This treatment may exhibit a dual benefit in both inhibiting viral attachment and reducing the host inflammatory response (Ou et al., 2020; Moore & June, 2020). Further research into the role of extracellular sialic acids in SARS-CoV2 attachment is necessary to fully clarify the role of Gal-3 inhibition as antiviral therapy.

## Supplemental Information

10.7717/peerj.9392/supp-1 Supplemental Information 1 Search strategy for our literature review.

## Additional Information and Declarations

### Data Availability

The following information was supplied regarding data availability: There is no raw data as this is a literature review.
