# Supplementary material for: A potential role for Galectin-3 inhibitors in the treatment of COVID-19
Source: PeerJ. 2020 Jun 15;8:e9392. doi: 10.7717/peerj.9392 (PMC7301894; doi:10.7717/peerj.9392)
Supplement: Supplemental Information 1 [file peerj-08-9392-s001.docx]

**Supplementary Table 1.** Search strategy for our literature review.

| Database | Search Queries |
| --- | --- |
| PubMed | **On SARS-CoV2**: ‘COVID-19 symptoms’ ‘SARS-CoV2 AND cytokine release syndrome’ ‘SARS-CoV2 entry mechanism’ ‘SARS-CoV2 AND galectins’ ‘SARS-CoV2 S1-NTD’ ‘SARS-CoV2 spike protein’ ‘SARS-CoV2 neurological symptoms’  **On β-coronaviruses**: ‘MERS-CoV entry mechanism’ ‘BCoV entry mechanism’ ‘SARS-CoV entry mechanism’ ‘coronavirus AND galectins’  **On Galectin-3**: ‘Galectins’ ‘Galectin-3’ ‘Galectin-3 AND cytokines’ ‘Galectin-3 AND inflammation’ ‘Galectin-3 AND viruses’ ‘Galectin-3 AND viral infection’  **On Galectin-3 Inhibitors**: ‘Galectin-3 inhibitors’ ‘TD139’ ‘belapectin’ ‘GR-MD-02’ |
| Google Scholar | **On SARS-CoV2**: ‘COVID-19,’ ‘COVID-19 symptoms’  **On Galectin-3:** ‘Galectins’ ‘Galectin-3 cytokines’  **On Galectin-3 Inhibitors:** ‘Galectin-3 inhibitors’ ‘GR-MD-02’ |
